# Orthoptera-TElib: a library of Orthoptera transposable elements for TE annotation

**DOI:** 10.1186/s13100-024-00316-x

**Published:** 2024-03-15

**Authors:** Xuanzeng Liu, Lina Zhao, Muhammad Majid, Yuan Huang

**Affiliations:** College of Life Sciences, Shaanxi Normal University, Xi’an, China

**Keywords:** Transposable elements, Orthoptera genome, TE database, Dfam and repbase, De novo annotation

## Abstract

Transposable elements (TEs) are a major component of eukaryotic genomes and are present in almost all eukaryotic organisms. TEs are highly dynamic between and within species, which significantly affects the general applicability of the TE databases. Orthoptera is the only known group in the class Insecta with a significantly enlarged genome (0.93-21.48 Gb). When analyzing the large genome using the existing TE public database, the efficiency of TE annotation is not satisfactory. To address this limitation, it becomes imperative to continually update the available TE resource library and the need for an Orthoptera-specific library as more insect genomes are publicly available. Here, we used the complete genome data of 12 Orthoptera species to de novo annotate TEs, then manually re-annotate the unclassified TEs to construct a non-redundant Orthoptera-specific TE library: Orthoptera-TElib. Orthoptera-TElib contains 24,021 TE entries including the re-annotated results of 13,964 unknown TEs. The naming of TE entries in Orthoptera-TElib adopts the same naming as RepeatMasker and Dfam and is encoded as the three-level form of “level1/level2-level3”. Orthoptera-TElib can be directly used as an input reference database and is compatible with mainstream repetitive sequence analysis software such as RepeatMasker and dnaPipeTE. When analyzing TEs of Orthoptera species, Orthoptera-TElib performs better TE annotation as compared to Dfam and Repbase regardless of using low-coverage sequencing or genome assembly data. The most improved TE annotation result is *Angaracris rhodopa*, which has increased from 7.89% of the genome to 53.28%. Finally, Orthoptera-TElib is stored in Sqlite3 for the convenience of data updates and user access.

**Supplementary Information:**

The online version contains supplementary material available at 10.1186/s13100-024-00316-x.

## Introduction

Transposable elements (TEs) are major components of eukaryotic genomes [1, 2], come in various forms and shapes [2], and have the ability to mobile and replicate themselves within genomes [3–5]. Recent studies found that TEs exist in almost all eukaryotes [6–8]. For example, more than 45% of the human genome consists of TEs [9]. In the plant kingdom, TEs cover 82.2% and between 85-90% of the wheat and maize genomes respectively [10–12]. In the fungal kingdom, TE content is less than 30% of the genome [13], and only 3% of TEs are in yeast genomes [14]. The TEs are highly variable within Insecta taxa [1, 15]; the genomic portion of TEs ranges from 2% in the *Belgica antarctica* (Diptera) [16] to 65% in the *Locusta migratoria* (Orthoptera) [17] and covers up to 75% of the genome of Vandiemenella viatical (Orthoptera). TEs are highly dynamic between and within species, studies of Orthoptera showed that most of the TEs are unique to each other in *Lo*cu*sta migratoria* and *Angaracris rhodopa* [18]. In Diptera for example, it’s rare to find shared TEs between the two species *Drosophila melanogaster* and *Drosophila simulans* [19, 20].

Repeated regions in the genome usually evolve much faster than single-copy DNA sequences [21], as well as the diversity and high dynamics of TEs [18], which greatly increase the difficulty of TE database construction and the bias of TE databases. In addition, there is no uniform classification system and nomenclature for TEs [22]. Finnegan (1989) proposed that TEs can be divided into two classes based on their transposition mechanisms: class I elements (retrotransposons) that transpose by reverse transcription using a DNA-RNA–DNA mechanism, and the class II elements (DNA transposons) transpose directly from DNA to DNA [23]. Both “Kapitonov and Jurka” (Repbase) and “Wicker” proposals retain the concept that all eukaryotic TEs in the original “Finnegan” proposal can be classified as retrotransposons or DNA transposons. The names of the division levels in the two proposals are different [6, 24–26]. The three levels in the Repbase proposal are called “type-class-superfamily” while they are called “class-order-superfamily” in the Wicker proposal. Among them, retrotransposons were classified as class I in the Wicker proposal and classified as Type 2 in the Repbase proposal. In Repbase, retrotransposons contain long terminal repeats at both ends (LTR retrotransposons) and lack LTRs (non-LTR retrotransposons) which includes both the long and short interspersed nuclear elements (LINEs and SINEs) as well as the Penelope-like elements [22, 27, 28]. The classification system of the Dfam database is also different from that of Repbase, which does not display a ranked hierarchy [29]. There are still no satisfying definitions for what a class, order, superfamily, or family of TEs constitutes.

RepBase and Dfam are commonly used reference databases for TE annotation, and both can be used together with RepeatMasker to identify repetitive sequences by searching genome-wide for sequences homologous to sequences present in the database [24, 25, 29–31]. Currently, the class Insecta only contains less than 18,000 TE entries in Repbase [25]. Using database-based homologous alignment for TE annotation can lead to bias between orders in Insecta due to the uneven distribution of TE consensus sequences in Repbase. In addition, when using low-coverage sequencing data for TE analysis, reference database selection also influences TE annotation results. A study on grasshoppers showed that when using dnaPipeTE software to annotate TE [18, 32], using the self-constructed TE library as a reference database in the -RM_LIB parameter, the annotation results were better than those of the public database (RepeatMasker.lib). Orthoptera is the only known group in the Insecta class with a significantly enlarged genome [33, 34], ranging from 0.93 Gb to 21.48 Gb [35]. The exploration of TEs in Orthoptera has been increasingly capturing the attention of researchers [18, 36]. The large genomes of Orthoptera insects pose a challenge as there is limited availability of genome assembly resources for this group. Moreover, most TE studies conducted on Orthoptera insects rely on low-coverage sequencing reads. Consequently, TE annotation heavily depends upon the choice of a reference database, influencing both the efficiency of TE annotation and the potential bias observed between species. These factors highlight the pressing need for an Orthoptera-specific TE library among researchers.

Here, we used the genome assembly data of 12 Orthoptera species that are available in NCBI to de novo annotate TEs with RepeatModeler2. The twelve species selected for this study represent four distinct families of Orthoptera (the detailed species list is in Table 1). We merged the TE libraries of these species to construct a non-redundant Orthoptera TE library (Orthoptera-TElib). A large number of unknown sequences in the TE library have been re-annotated. In addition, we refer to the naming rules (level 1/ level 2-level 3) of Repeatmasker.lib and Dfam. We evaluated the performance of the Orthoptera TE library (Orthoptera-TElib) and TE public database in TE annotation of Orthoptera species using RepeatMasker and dnaPipeTE software. Orthoptera-TElib was stored in Sqlite3 format, enabling convenient data updates and user access.

**Table 1:** The List of 12 Orthoptera species and TE libraries built by RepeatModeler2

Species	family	GenBank assembly accession	TE libraries built by RepeatModeler2
***Locusta migratoria***	Acrididae	GCA_026315105.1	Total: 4441, Type1: 867, Type2: 1209, Unknown 2365
***Schistocerca gregaria***	Acrididae	GCA_023897955.2	Total: 2990, Type1: 686, Type2: 572, Unknown: 1732
***Schistocerca americana***	Acrididae	GCA_021461395.2	Total: 3269, Type1: 685, Type2: 615, Unknown: 1969
***Schistocerca nitens***	Acrididae	GCA_023898315.2	Total: 3256, Type1: 727, Type2: 612, Unknown: 1917
***Schistocerca cancellata***	Acrididae	GCA_023864275.2	Total: 3756, Type1: 758, Type2: 1230, Unknown: 1768
***Schistocerca piceifrons***	Acrididae	GCA_021461385.2	Total: 3248, Type1: 714, Type2: 621, Unknown: 1913
***Schistocerca serialis cubense***	Acrididae	GCA_023864345.3	Total: 3174, Type1: 706, Type2: 595, Unknown: 1873
***Gryllus bimaculatus***	Gryllidae	GCA_017312745.1	Total: 2409, Type1: 348, Type2: 353, Unknown: 1708
***Teleogryllus occipitalis***	Gryllidae	GCA_011170035.1	Total: 2965, Type1: 441, Type2: 557, Unknown: 1967
***Laupala kohalensis***	Gryllidae	GCA_002313205.1	Total: 3399, Type1: 364, Type2: 771, Unknown: 2264
***Meconema thalassinum***	Tettigoniidae	GCA_946902985.1	Total: 3326, Type1: 607, Type2: 560, Unknown: 2159
***Xya riparia***	Tridactylidae	New assembly (https://doi.org/10.6084/m9.figshare.19336391.v1)	Total: 2580, Type1: 572, Type2: 755, Unknown: 1253
		**Total: 38813**	Type1 7475, Type2 8450, Unknown 22888

## Results

### De novo transposable element (TE) identification and Orthoptera TE library construction

**Fig. 1:**
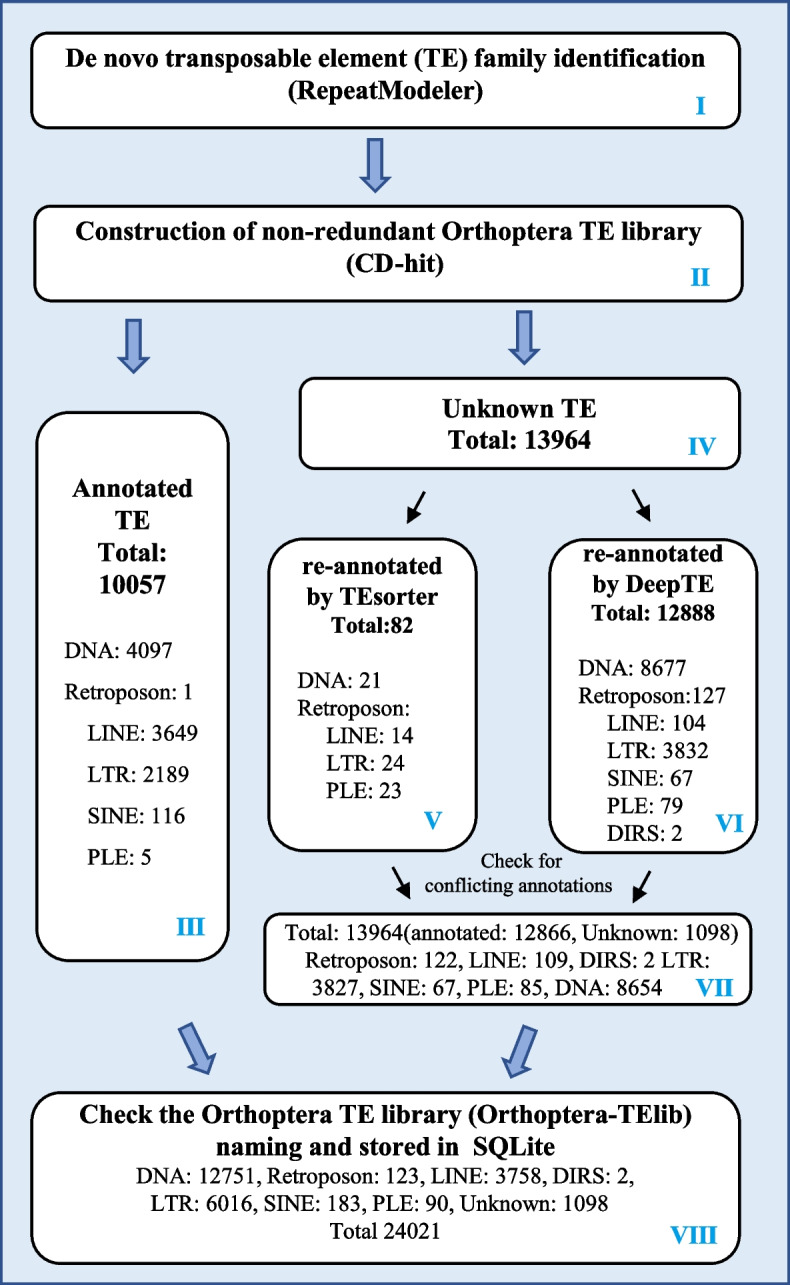
Orthoptera-TElib build flowchart. DNA: DNA transposons. The box VI “Retroposon: 127” means that 127 TE entries were identified as retrotransposons but not classified at the “class” level. In box VI “Retroposon: 127” and in box VII “Retroposon: 122”, this situation occurs because some of the 127 TE entries recognized as "Retroposon" by DeepTE are further classified by TEsorter. The annotation information of each TE entry in boxes III, V, VI, VII, and VIII were uploaded to Supplementary Table S1-S5

We performed de novo annotation of repetitive sequences for 12 Orthoptera species using available genome assembly data. These species belong to Acrididae, Tettigoniidae, Gryllidae, and Tridactylidae families, exhibiting varying genome sizes ranging from 1.595 Gb in *Laupala kohalensis* to 9.083 Gb in *Schistocerca serialis cubeense*. We used RepeatModeler2 to generate 12 repeat libraries (see Methods). RepeatModeler2 can generate high-quality TE family libraries suitable for use with RepeatMasker and final submission to the Dfam database. The repeated sequence library we obtained refers to the classification system of Dfam and the naming of the sequence conforms to the input standard of RepeatMasker. This standard will also serve as the naming convention for TE sequences in Orthoptera-TElib. The initial library contains other types of repetitive elements besides TEs, which are not included in our statistics, such as satellite DNA. Among the TE libraries of 12 species (Table 1), the species with the most entries is *Locusta migratoria*, which contains 4441 TE sequences, including 867 DNA transposons, 1209 retrotransposons, and 2365 unknown TE sequences. The TE libraries of 12 species were merged into a preliminary Orthoptera TE library (Orthoptera-TElib). This preliminary Orthoptera-TElib comprises a total of 38,813 sequences, which includes 7475 DNA transposons, 8450 retrotransposons, and 22,888 unknown TE sequences. To avoid redundant sequences in the merged TE library, we used CD-hit to remove redundancy (see Methods). A total of 24,021 sequences were obtained in the non-redundant Orthoptera TE library, including 10,057 classified TE sequences and 13,964 unannotated TE sequences. The preliminary Orthoptera-TElib contains a large number of unknown TEs, and these sequences could affect the annotation efficiency when using Orthoptera-TElib as a reference library. We have re-annotated these unknown TEs and checked the naming to build a complete Orthoptera-TElib. The detailed annotation process is shown in Fig. 1. Orthoptera-TElib uses the form of level 1/ level 2-level 3 to name TE entries. Level 2 corresponds to the superfamily-level of Repbase, and level 3 corresponds to the family-level. It is worth noting that level 1 corresponds to the type-level of Repbase for DNA transposons and the class-level of Repbase for retrotransposons (e.g. “LINE/RTE-BovB” and “DNA/hAT-Charlie”).

To annotate 13,964 unknown TEs, we used the DeepTE based on the Convolutional Neural Network and the classification software TEsorter based on the Hidden Markov Model (see Methods). The TEsorter only annotated 82 out of the 13,964 unknown TEs (Fig. 1 box V and Supplementary Table S1). TEsorter is more effective in classifying known TEs than re-annotating unknown TE sequences. DeepTE annotated 12,888 out of 13,964 unknown TE sequences, including 8677 DNA transposons and 4211 retrotransposons (Fig. 1 box VI and Supplementary Table S2). There are 77 TE entries that were re-annotated by both TEsorter and DeepTE (Supplementary Table S6). However, there are conflicts in the annotation results of 45 TE sequences in the two software. For example, “id5353_rnd-1__Meconema_thalassinum-116” is annotated as “LTR/Gypsy” in DeepTE and as “DNA/Maverick” in TEsorter. For annotation conflicts of 45 TE entries, we performed additional detection using TEclass2 based on machine model Transformer and Domain Based ANnotation of Transposable Elements (DANTE) (see Methods). DANTE annotated 13 TE entries (Supplementary Table S7) and TEclass2 annotated 20 TE entries (Supplementary Table S8). We retained annotations for 18 TE entries with at least two identical evidences, and the 37 remaining conflicting annotation entries were recorded as “Unknown TEs” (Supplementary Table S9). We combined the annotation results of the two software, which annotated 8654 DNA transposons, 122 named retrotransposons, 109 LINEs, 3827 LTRs, 67 SINEs, 85 PLEs, and 2 DIRSs (Fig. 1 box VII). After 13,964 unknown TE sequences were re-annotated, 12,866 TE entries were annotated and 1,098 sequences remained unannotated. To be clear, not every TE entry will be classified to level 3 when de novo annotated. Some TE entries are annotated to level 2 or level 1, and they are encoded as level 1/ level 2 (e.g. “DNA/ hAT”, “LINE/ RTE”) or level 1 (e.g. “DNA”, “Retroposon”) in OTElib. The TE entry named “Retroposon”, which was identified as retrotransposon but not classified at the “class” level. The complete Orthoptera-TElib is obtained by merging the 10,057 TE entries annotated from the beginning by RepeatModeler2 and the re-annotated results of 13,964 TE entries (annotated 12,866 TE entries and unclassified 1,098 TE entries). Orthoptera-TElib contains 24,021 TE entries, and the current number has exceeded the TE entries of Insecta in Repbase.

### Orthoptera-TElib classification standard and naming rules

To facilitate the storage of TE libraries in the database, we defined the classification of Orthoptera-TElib into four levels: Type-Class-superfamily-family. At the first level, all TEs are classified into Type 1 (DNA transposons) and Type 2 (retrotransposons) elements. At the second level of classification of DNA transposons, we divided DNA transposons into four classes (Transposase, DNA polymerase, Tyrosine recombinase, and Helicase) according to the characteristics of the enzymes contained in the elements. We classified retrotransposons into five classes (LINE, LTR, SINE, PLE and DIRS) in Orthoptera-TElib. Some TE superfamilies contained in the Repbase and Dfam databases are not present in Orthoptera, and we did not record these superfamilies in Orthoptera-TElib, such as DIRS/Ngaro. The de novo annotation results of RepeatModeler2 and DeepTE conform to the Dfam database standard, so the Orthoptera-TElib classification system refers to the Dfam standard. The Orthoptera-TElib contains 39 TE superfamilies (Table 2). The Orthoptera-TElib contains 39 TE superfamilies (Table 2). Since some TE entries are only classified into “superfamily” level or “class” level, we do not count family “level” annotation information. The Orthoptera-TElib preserves the annotation results at the “family” level and the classification information of each TE entry can be viewed in Supplementary Table S5.

**Table 2:** Orthoptera-TElib classification standards

Type	Class	Superfamily	Orthoptera-TElib name
**DNA transposons**	Transposase	Tc1-Mariner	DNA/ Tc1-Mariner
		hAT	DNA/hAT
		Mutato	DNA/Mutato
		Merlin	DNA/Merlin
		P	DNA/P
		PiggyBac	DNA/PiggyBac
		PIF	DNA/PIF
		Harbinger	DNA/Harbinger
		Sola	DNA/Sola
		Academ	DNA/Academ
		CACTA	DNA/CACTA
		Ginger	DNA/Ginger
		Kolobok	DNA/Kolobok
		Zator	DNA/Zator
		Zisupton	DNA/Zisupton
	DNA polymerase	Maverick	DNA/Maverick
	Tyrosine_Recombinase	Crypton	DNA/Crypton
	Helicase	Helitron	RC/Helitron
**Retrotransposons**	LINE	CR1	LINE/CR1
		Dong	LINE/Dong
		I	LINE/I
		Jockey	LINE/Jockey
		L1, L2	LINE/L1, L2
		R1, R2	LINE/R1, R2
		RTE	LINE/RTE
		Rex	LINE/Rex
		Tad1	LINE/Tad1
	LTR	Bel-Pao	LTR/Bel-Pao
		Copia	LTR/Copia
		Gypsy	LTR/Gypsy
		ERV	LTR/ERV
	PLE	Chlamys	PLE/Chlamys
		Naiad	PLE/Naiad
	SINE	Alu	SINE/Alu
		MIR	SINE/MIR
		U	SINE/U
		5 s	SINE/5 s
		tRNA	tRNA
	DIRS	DIRS	DIRS

Our original intention in establishing Orthoptera-TElib is that it can be used as an input reference database for repetitive sequence analysis software. The current mainstream repeat sequence analysis software, RepeatMasker for genome assembly data and dnaPipeTE for low-coverage sequencing data, rely on the input library formatted according to the RepeatMasker.lib naming convention. Therefore, the naming of TE entries in Orthoptera-TElib is checked according to the rules in RepeatMasker.lib. The encoding pattern (“level 1/level 2-level 3”) of TE entries in Orthoptera-TElib conforms to the input format of the reference database for repetitive sequence analysis software. It should be noted that this naming convention is different for DNA transposons and retrotransposons at level 1. For DNA transposons, level 1 is coded as “DNA”; for retrotransposons, level 1 is coded as Class levels in Orthoptera-TElib, such as LINE, LTR, and SINE. The naming rules of level 2 and level 3 are consistent in DNA transposons and retrotransposons, level 2 is encoded as the superfamily level in Orthoptera-TElib, and level 3 is encoded as the family level.

### Application and efficiency of Orthoptera-TElib

**Fig. 2:**
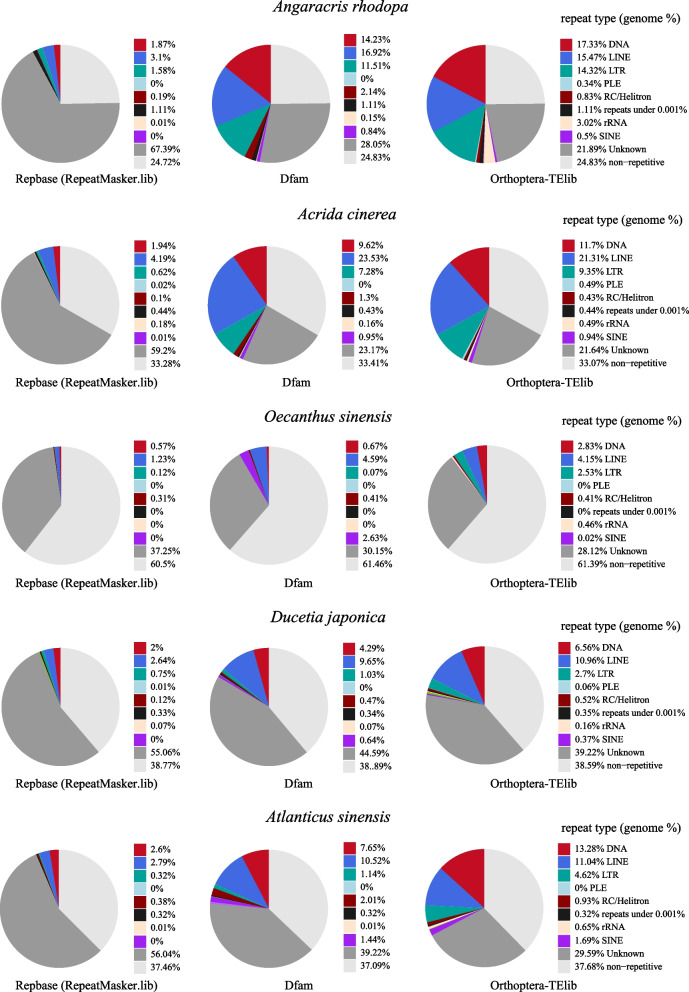
The performance of Orthoptera-TElib as a reference database during TE annotation. A total of five orthoptera species were chosen to compare reference databases using the dnaPipeTE software. The annotation results for TEs with the default Repbase (RepeatMasker.lib) reference database are presented in the pie chart on the left, the pie chart in the middle is the annotation result using Dfam as the reference data, and the pie chart on the right side displays the results obtained from the Orthoptera-TElib database. Notably, the legend of the charts does not include simple repeats and tRNAs found in the genome. Detailed repeat analysis results are shown in Supplementary Figure S1

We first tested the performance of Orthoptera-TElib using low-coverage sequencing reads from five Orthoptera species (*Angaracris rhodopa, Acrida cinerea, Oecanthus sinensis, Ducetia japonica, and Atlanticus sinensis*) (genome sizes from 1.06–16.00 Gb) [18, 34, 37] by using dnaPipeTE to compare the differences between the three reference databases (Repbase, Dfam, and Orthoptera-TElib). The TE analysis results of the five species revealed a substantial presence of unannotated unknown TEs utilizing the default database, Repbase (RepeatMasker.lib). The repetitive sequences identified in these species constituted a significant proportion of the genome, ranging from 39.5% to 75.28%. Due to the presence of a large number of unannotated repetitive sequences (as “Unknown” in Fig. 2) in the analysis results, the annotated TEs accounted for only a small fraction, specifically ranging from 2.25% to 7.89%. The species with the most unknown TE was *Angaracris rhodopa*, which reached 67.39% of the genome. The annotation results of DNA transposons only account for 0.57%-2.6% of the genome, the genome proportion of LTR was 0.12%-1.58%, and the genome proportion of LINE ranged from 1.23% to 4.19%. The annotation results of other TEs are shown in Fig. 2. When Dfam is used as the reference database, the annotation efficiency of TE is improved compared with Repbase. The content of repetitive sequences in the genomes of the five species ranged from 38.54% to 75.17%, and the annotated TEs accounted for 8.39% to 45.64% of the genomes (Fig. 2).

When Orthoptera-TElib is selected as the reference database, the proportion of repetitive sequences has hardly changed compared to the use of Repbase and Dfam, accounting for 75.17%-38.61% of the genome. However, the annotation efficiency of TE has been significantly improved compared with the previous two TE databases, the annotated TE accounting for 10.49% (*O. sinensis*)-53.28% (*A. rhodopa*) of the genome (Fig. 2). Among them, the most improved annotation result is *A. rhodopa*, which has increased from 7.89% of the genome to 53.28%. The DNA transposon annotation results show that *O. sinensis* had the lowest content, which increased from 0.57% to 2.83%. For DNA transposon annotation, *A. rhodopa* had the highest content, which increased from 1.87% to 17.33% in terms of genome proportion. The annotation results of other TE classes have also improved significantly (Fig. 2). In general, Orthoptera-TElib as a reference database can significantly increase the annotation efficiency of TE in Orthoptera species when using low sequencing depth reads to analyze repetitive sequences.

We also evaluated the performance of Orthoptera-TElib using it as a custom library of RepeatMasker for genome masking. To run RepeatMasker, the input usually involves the RepeatModeler library-a repeat sequence library constructed from the genome of the respective species. We merged the library constructed by RepeatModeler2 with Orthoptera-TElib (hereafter referred to as the merged library) and used it as the input library of RepeatMasker to mask the genome. The *Xya riparia*, *Gryllus bimaculatus*, and *Laupala kohalensis* genomes were used to test the masking efficiency of the two libraries (RepeatModeler library and merged library). The total chromosome length of *Xya riparia* is 1,583,593,013 bp and a total of 701,392,827 bp (Repeat sequence accounting for 44.29% of the genome) was masked when the RepetModeler library was used to mask the genome (Supplementary Table S10). A total of 714,158,129 bp (Repeat sequence accounting for 45.10% of the genome) were masked when the merged library was used to mask the genome (Supplementary Table S11). The annotation results of the two methods differ significantly in the content of unclassified TE. When the RepeatModeler library is used, the unclassified TE accounts for 16.25% of the genome (Supplementary Table S10), while the unclassified TE accounts for only 1.52% of the genome in the result of the merged library (Supplementary Table S11). The DNA transposons annotated by the former accounted for 8.85% of the genome and retrotransposons accounted for 17.41% of the genome, while the latter annotated DNA transposons accounted for 20.85% of the genome and retrotransposons accounted for 20.85% of the genome. In addition, Orthoptera-TElib also greatly improved the annotation of TEs in the *G. bimaculatus* genome, with unclassified repetitive sequences decreasing from 22.92% to 1.23% of the genome (Supplementary Table S12-S13). Similarly, Orthoptera-TElib also improved TE annotation in the *L. kohalensis* genome (unclassified repetitive sequences decreased from 25.22% to 1.82% of the genome) (Supplementary Table S14-S15). It is evident that the introduction of Orthoptera-TElib did not cause bias in the evaluation of the content of genomic repeat sequences but greatly reduced the content of Unclassified TE in the results.

Finally, we used Sqlite3 to generate Orthoptera-TElib.db (SQL format) from Orthoptera-TElib (fasta format) to facilitate data updates. The table Orthoptera-TElib is created in Orthoptera-TElib.db, which contains five fields: unique id, species name, TE Class, TE superfamily, and sequence. Users can search the required sequence according to the species name, TE class, and TE superfamily generating Fasta format files. For example, users can run SQL (Structured Query Language) " SELECT * FROM OTElib WHERE class = 'LTR' " to obtain LTR records (Fasta format) in Orthoptera-TElib.

## Discussion

When analyzing TEs of Orthoptera species, Orthoptera-TElib performs satisfactorily regardless of using low-coverage sequencing or genome assembly data. Orthoptera-TElib will not affect the determination of the repetitive sequence contained within the genome. Its purpose is solely to enhance the effectiveness of TE class or superfamily annotation. Secondly, using Orthoptera-TElib as a reference database can more accurately reflect the content of a certain type of TE in a species. If the choice of reference database is unreasonable, it will lead to wrong conclusions when comparing the content of a certain type of TE in two species. For example, when comparing repeat sequences in *Angaracris rhodopa* and *Acrida cinerea*, if Repbase (RepeatMasker.lib) is used as a reference database, the genome proportion of DNA transposons in *A. rhodopa* is 1.87% which is less than that in *Acrida cinerea* representing 1.94% (Fig. 2). When the reference database was changed to Orthoptera-TElib, the genome proportion of DNA transposons in *A. rhodopa* significantly increased to 17.33%, surpassing the 11.7% observed in the *Acrida cinerea* (Fig. 2). Finally, when analyzing TE with low-coverage sequencing reads, using Orthoptera-TElib as a reference database obtained a higher number of annotated TE consensus sequences in the results. A total of 6,563 annotated TE consensus sequences were obtained for *A. rhodopa* when RepeatMasker.lib was used, whereas 56,356 annotated TE consensus sequences were obtained when Orthoptera-TElib was used. This indicates a considerable advantage of using Orthoptera-TElib for TE analysis in getting more annotated TE consensus sequences.

RepeatModeler2 was used in the de novo TE annotation of the Orthoptera genome during the construction of Orthoptera-TElib. A study on software evaluation for de novo detection of transposons showed that RepeatModeler beats competitors (RepeatScout and REPET) in most datasets [38]. Other annotation software also did not perform well when further classifying unclassified TEs in RepeatModeler results. We used TEsorter and DeepTE to re-annotate these unclassified TEs and the annotation results of 45 TE entries were conflicting. When using additional software to check the conflicting annotation results, some of the annotation results of TEclass2 are consistent with TEsorter and some are consistent with DeepTE, while the annotation results of DANTE are all consistent with those of TEsorter (Supplementary Table S9). Both DANTE and TEsorter use a TE protein domain-based method, which may be more accurate when annotating TEs. In a study that used four software (TEsorter, RepeatClassifier, DeepTE, and TERL) to annotate rice genome TEs, it was found that TEsorter had the highest precision and DeepTE had the highest sensitivity [39].

The debate over the TE classification system has always continued; no one proposal provides a satisfactory implementation of a proper scientific classification at all levels. The proposals of Finnegan, Wicker, and Repbase may be more suitable for classification at and below the superfamily level due to their emphasis on sequence similarity [6, 23, 24]. The classification system of TE in Orthoptera-TElib is similar to that of Dfam because the results generated by the TE annotation software (RepeatModeler2, DeepTE, and TEsorter) are directly applicable to the Dfam database, and this TE library is also suitable for the input reference database of RepeatMasker and dnaPipeTE. The Dfam classification system does not show an order hierarchy, whereas we define the classification system of Orthoptera-TElib into four levels: Type-Class-superfamliy-family. It is worth noting that in the classification of DNA transposons, Orthoptera-TElib uses the characteristics of enzymes to name at the class level, which is consistent with Dfam. This classification standard is similar to Repbase’s proposal, although there are variations in naming. DNA transposons containing transposases are called terminal inverted repeats (TIRs) at the class-level in Repbase, while Orthoptera-TElib directly uses “transposases” to name them. TEs have evolved from numerous transposition mechanisms with independent origins. The classification standards among TE databases need to be more consistent, and different TE analysis software has different naming rules for reference databases and output results. We need an international committee to standardize the TE classification system [22].

The annotation method based on homologous sequence alignment has a bias in the annotation results due to the close relationship between the database and the analyzed species. This bias may also occur with Orthoptera-TElib, which has a more efficient annotation of repetitive sequences when analyzing species closely related to Orthoptera-TElib. We found that when using Orthoptera-TElib to annotate the TE of the *Ducetia japonica* genome, although it has improved compared to the results using the Dfam and Repbase databases, there are still a large number of unknown TEs accounting for 39.22% of the genome. The result of using Orthoptera-TElib to annotate the TE of Acrididae is better than that of Tettigoniidae. Orthoptera is the only known group in the Insecta class with a significantly enlarged genome, it is crucial to expand the repertoire of TEs in Orthoptera-TElib by incorporating species from different families to mitigate biases. We provide a Python script to store new records in Orthoptera-TElib.db (https://github.com/Liuxuanzeng/OTElib), and we encourage users to upload the TE consensus sequences of Orthoptera insects found to Orthoptera-TElib.db.

## Materials and methods

### Materials, DNA extraction, and sequencing

The genome assembly data of *Xya riparia* was downloaded from the figshare (https://doi.org/10.6084/m9.figshare.19336391.v1) and the genome assembly data of the other 11 species’ genome assembly data were downloaded from NCBI (the GenBank assembly accession numbers are listed in Table 1).

The Raw genome sequencing data of the *A. rhodopa* was downloaded from NCBI SRA (SRR19352342). Live adults of *Acrida cinerea*, *Oecanthus sinensis*, *Ducetia japonica*, and *Atlanticus sinensis* were taken to the laboratory for dissection. The samples were added to 95% ethanol and stored in a − 20 °C freezer. We extracted the genomic DNA of *Acrida cinerea*, *Oecanthus sinensis*, *Ducetia japonica*, and *Atlanticus sinensis* from the hind leg of one female using an SDS-based lysis method and purified the DNA with chloroform. The extracted DNA was sonicated to a fragment size of 350 bp. The library was fixed onto a microarray by bridge PCR and sequenced using the Illumina HiSeq 2500 sequencing platform (PE150bp).

### De novo transposable element (TE) family identification

RepeatModeler2 for the automated genomic discovery of transposable element families (https://github.com/Dfam-consortium/TETools) was used for de novo annotation. Genomes of 12 Orthoptera species were used as input to identify TE families. First, the BuildDatabase command in RepeatModeler2 was run to build the genome index (BuildDatabase -name Speciesname genome.fa). Secondly, TE de novo annotation was performed to construct the transposon library (RepeatModeler -database Speciesname -threads 64 -LTRStruct). The TEs annotated in the transposon library built by RepeatModeler adopt a three-level naming form (level1/level2-level3), and the unannotated TEs are represented by “Unknown”.

### Construction of non-redundant Orthoptera TE library

First, we merged the TE libraries of 12 species into one Orthoptera TE library (cat *.lib > 12species_TEfamilies.lib). Second, redundant sequences in the combined TE library were removed. Wicker et al. proposed to define a family as a group of TEs that can be aligned over at least 80 bp and show 80% + identity covering 80% or more of the alignment [6]. In this step, redundant sequences need to be removed from the merged TE entries of the 12 species, we used CD-hit (https://github.com/weizhongli/cdhit) to remove redundant sequences using the 80–80-80 principle (cd-hit-est -i 12species_TEfamilies.lib -o 12species_ TEfamilies_nr08.lib -d 0 -aS 0.8 -c 0.8 -G 0 -g 1 -b 500 -T 0 -M 256000).

### Unknown TE re-annotated

We first used seqkt to extract the unknown TE sequences in Orthoptera-TElib (https://github.com/lh3/seqtk)(seqtk subseq Orthoptera-TElib.fa unknown_name > out.fa). In the next step, unknown TEs were re-annotated using TEsorter (https://github.com/zhangrengang/TEsorter) [39] and DeepTE (https://github.com/LiLabAtVT/DeepTE). DeepTE is aimed to classify transposons with unknown classification via Convolutional Neural Network (python3 DeepTE.py -o output_dir -d workpath -i 12species_unknownTE.fa -m_dir Metazoans_model/ -sp M). We also use TEsorter to re-annotate unclassified TEs and REXdb-metazoa as reference databases [40] (TEsorter 12species_unknownTE.fa -db rexdb-metazoa -p 64). We refer to the annotation results of the two software and rename these reannotated unknown TEs in Orthoptera-TElib.

### DANTE and TEclass2 re-annotated 45 TE entries with conflicting annotations

The annotation of TE entries was done through Domain Based Annotation of Transposable Elements (DANTE) (https://repeatexplorer-elixir.cerit-sc.cz/galaxy). We choose the taxon and protein domain database version as REXdb (Metazoa_version_3.1) [40]. We re-annotated the conflicting TEs using the online version of TEclass2, requiring only a Fasta file as input data (https://github.com/IOB-Muenster/TEclass2) (https://www.compgen.uni-muenster.de/tools/teclass/generate/).

### Store Orthoptera-TElib in sqlite3

We used a Python script to generate a SQLite3 database of Orthoptera-TElib.db (https://github.com/Liuxuanzeng/OTElib). The table Orthoptera-TElib is created in Orthoptera-TElib.db, which contains five fields: unique id, species name, TE Class, TE superfamily, and sequence. Users can use our provided Python script to update Orthoptera-TElib.db.

### Evaluate the performance of Orthoptera-TElib for Orthoptera TE annotation

dnapipeTE was used to analyze genomic repeats from low-coverage sequencing reads (https://github.com/clemgoub/dnaPipeTE). We used 0.1 × genome coverage sequencing reads as input data Repeatmasker.lib and Orthoptera-TElib as reference databases to compare the results of genome TE annotation. The dnaPipeTE software installation and operation are as follows (sudo docker pull clemgoub/dnapipete:latest) (python3 dnaPipeTE.py -input sequencing.fq.gz -output output_dir -RM_lib Orthoptera-TElib -genome_size -genome_coverage 0.1 -sample_number 2 -RM_t 0.3 -contig_length 350 -cpu 32). We use the dnaPT_charts.sh script to generate pie charts of the proportion of repeats (https://github.com/clemgoub/dnaPT_utils) (dnaPT_charts.sh -I dnaPipeTE.OUT -p output_name -o ouput_dir -t 0.0001).

In this step, RepeatMasker was used to evaluate the annotation efficiency of TEs in the genome (Orthoptera-TElib and RepeatModeler library). RepeatMasker was used to analyze genomic repeats using genome assembly data (http://repeatmasker.org). We used the library constructed by RepeatModeler2 and merged library (Orthoptera-TElib and RepeatModeler library) as the input of RepeatMasker to mask the genome of *Xya riparia* (RepeatMasker -pa 80 -html -gff -poly -lib merged_Orthoptera-TElib Xya_genome -dir output_dir).

### Supplementary Information

**Additional file 1: Table S1.** Re-annotated results by using TEsorter. (Figure 1 box V TE entries). **Table S2.** Re-annotated results by using DeepTE. (Figure 1 box VI TE entries). **Table S3.** Figure 1 box III TE entries. **Table S4.** Figure 1 box VII TE entries. **Table S5.** Annotation information for 24,021 TE entries in Orthoptera-TElib. (Figure 1 box VIII TE entries). **Table S6.** The 77 TE entries were annotated by both DeepTE and TEsorter. **Table S7.** DANTE re-annotation results of 45 conflicting TE entries. **Table S8.** TEclass2 re-annotation results of 45 conflicting TE entries. **Table S9.** The result of the final annotation of 45 conflicting TE entries. **Table S10.** Results of running RepeatMasker on the *Xya riparia* genome using the RepetModeler library. **Table S11.** Results of running RepeatMasker on the *Xya riparia* genome using the merged library. **Table S12.** Results of running RepeatMasker on the *Gryllus bimaculatus* genome using the RepetModeler library. **Table S13.** Results of running RepeatMasker on the *Gryllus bimaculatus* genome using the merged library. **Table S14.** Results of running RepeatMasker on the *Laupala kohalensis* genome using the RepetModeler library. **Table S15.** Results of running RepeatMasker on the *Laupala kohalensis* genome using the merged library.

**Additional file 2: Fig. S1.** The proportion of repetitive elements in the genomes of five species.

## Data Availability

The genome assembly data of 12 species come from figshare (https://doi.org/10.6084/m9.figshare.19336391.v1) and NCBI (the GenBank assembly accession numbers are listed in Table 1). Raw genome sequencing data of four Orthoptera species have been made publicly available through the NCBI Sequence Read Archive (PRJNA1007903). The Raw genome sequencing data of the *A. rhodopa* was downloaded from NCBI SRA (SRR19352342). Additionally, the Orthoptera-TElib.fa, Orthoptera-TElib.db, and the initial TE libraries for each species have been deposited into the figshare database (https://doi.org/10.6084/m9.figshare.23993616.v3).
