# Supplementary figures and images for: Orthoptera-TElib: a library of Orthoptera transposable elements for TE annotation
Source: Mob DNA. 2024 Mar 15;15:5. doi: 10.1186/s13100-024-00316-x (PMC10941475; doi:10.1186/s13100-024-00316-x)

Figure S1 The proportion of repetitive elements in the genomes of five species.

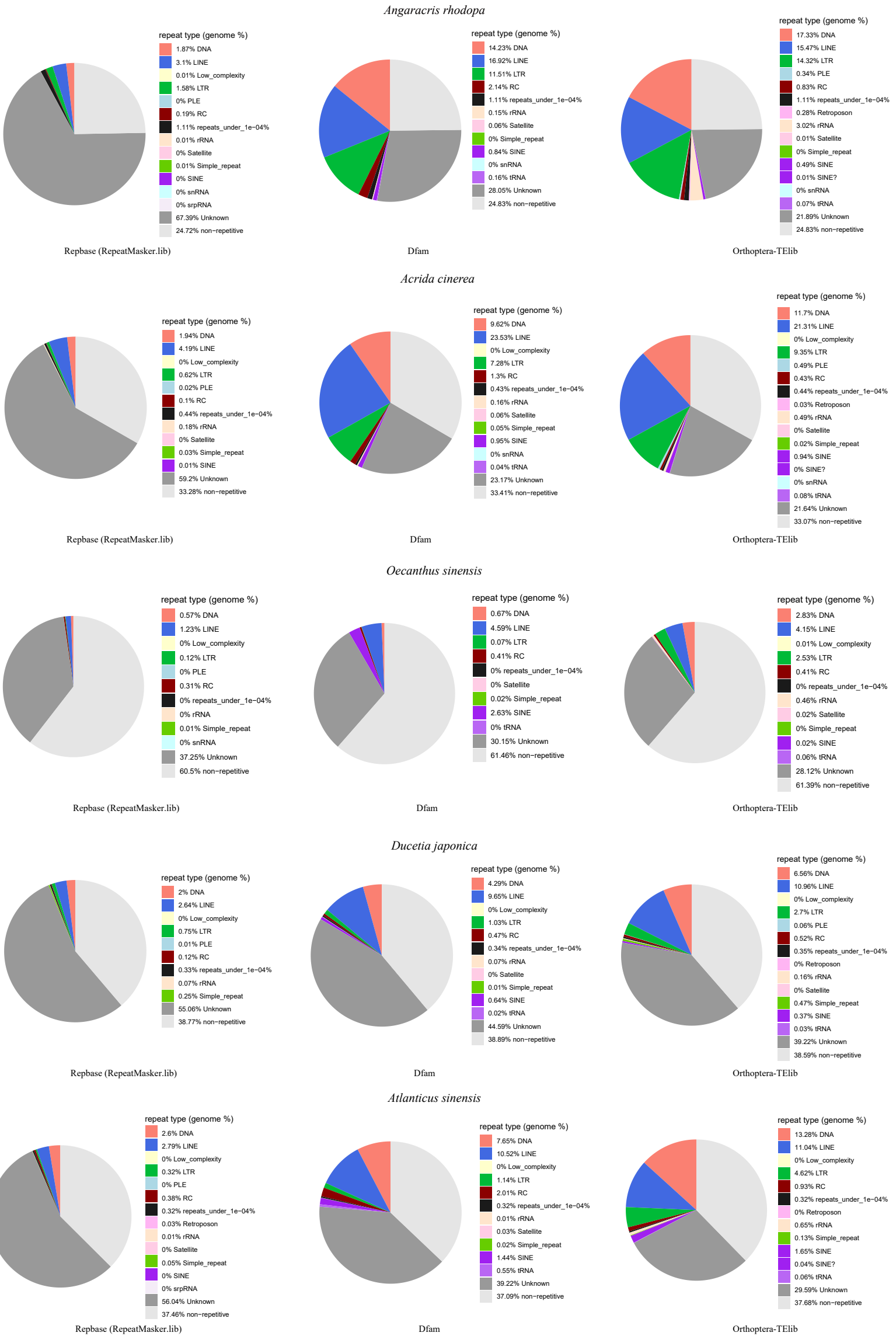

Supplement: Supplementary file 2 — Additional file 2: Fig. S1. The proportion of repetitive elements in the genomes of five species. [file 13100_2024_316_MOESM2_ESM.pdf]
